# Electronic Rearrangements and Angular Momentum Couplings in Quantum State-to-State Channels of Prototype Oxidation Processes

**DOI:** 10.1021/acs.jpca.0c09701

**Published:** 2021-02-16

**Authors:** Stefano Falcinelli, Franco Vecchiocattivi, Fernando Pirani

**Affiliations:** † Department of Civil and Environmental Engineering, University of Perugia, Via G. Duranti 93, 06125 Perugia, Italy; ‡ Department of Chemistry, Biology and Biotechnologies, University of Perugia, Via Elce di Sotto 8, 06123 Perugia, Italy

## Abstract

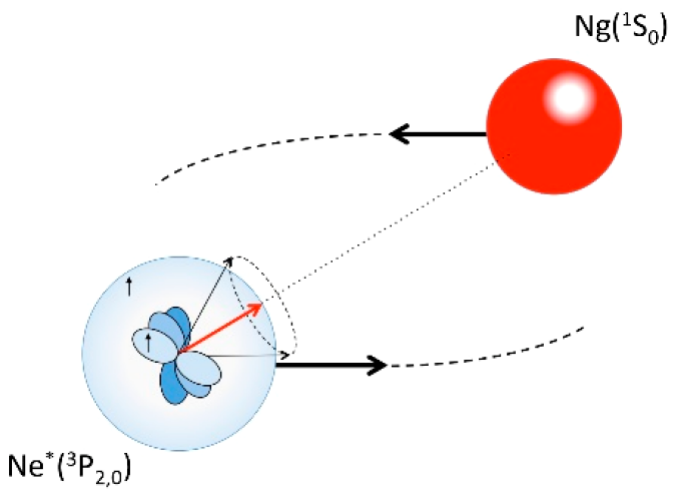

An innovative theoretical method to describe the microscopic dynamics of chemi-ionization reactions as prototype oxidation processes driven by selective electronic rearrangements has been recently published. It was developed and applied to reactions of Ne* atoms excited in their metastable ^3^P_J_ state, and here, its physical background is extensively described in order to provide a clear description of the microscopic phenomenon underlying the chemical reactivity of the oxidative processes under study. It overcomes theoretical models previously proposed and reproduces experimental results obtained in different laboratories. Two basic reaction mechanisms have been identified: (i) at low collision energies, a weakly bounded transition state is formed which spontaneously ionizes through a radiative physical mechanism (photoionization); (ii) in the hyperthermal regime, an elementary oxidation process occurs. In this paper, the selectivity of the electronic rearrangements triggering the two mechanisms has been related to the angular momentum couplings by Hund’s cases, casting further light on fundamental aspects of the reaction stereodynamics of general interest. The obtained results allow peculiar characteristics and differences of the terrestrial oxidizing chemistry compared to that of astrochemical environments to be highlighted.

## Introduction

Most chemical reactions occur via a multistep mechanism, and often, they are triggered by charge (electron) transfer (CT) phenomena. The traditional chemist, usually working in the liquid phase, distinguished them as acid–base, redox, and proton affinity reactions. Moreover, in acid–base (Lewis theory) and even in nucleophilic substitution SN1 and SN2 reactions (fundamental processes in organic chemistry), one or more electron pairs are exchanged or shared.

The study of gas phase elementary reactions, in the absence of the solvent and under single collision events, more directly highlighted the role of CT. In this regard, we can mention: i) the harpooning processes, as evidenced in the oxidation reactions between a metal atom (mainly an alkaline metal) and a species with high electron affinity^1−3^; ii) the Coulomb explosions of molecular dications^4,5^; iii) the formation of excimer compounds which are involved in powerful UV lasers^6,7^; iv) the chemi-ionization reactions (ChemI).^8−10^ Many studies focused on individual aspects of these phenomena, but still today, there is not a unifying description of them.

ChemI are elementary fast reactions stimulated by electronic rearrangements taking place within the collision complex (X···M)* (i.e., the transition state (TS) formed by an excited X* atom and an atomic/molecular species M as reagents). Their dynamical evolution is directly affected by the selectivity of CT, as shown below:
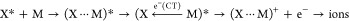
However, this represents a compact formulation providing only a schematic representation of a ChemI reaction. In particular, X* can be an anisotropic atom, as Ne*, the CT within the collision complex must depend on relative alignment/orientation of X* and M reagents, and finally, in addition to electrons, the other products can be associate XM^+^ and Penning M^+^ ions (including also possible fragmentations of M^+^) that can be formed both in the ground and in excited internal states.

Penning ionization electron spectroscopy, an important tool in the principal characterization of the collision complex which can be related to the TS of such reactions,^11^ combined with the extensive experimental and theoretical study of the collision dynamics, suggested using a new approach for the description at a microscopic level of these basic processes. It casts light on unknown aspects of their stereodynamics which are of general interest, since ChemI represent the competition of elementary chemical–oxidation and physical photoionization gas phase processes (see below).

ChemI are a prototype of barrier-less reactions whose detailed investigation is still today of great interest for fundamental and applied research. Indeed, they control the balance of phenomena that occur in interstellar environments, in combustion and flames (in which they are considered as the initial primary phase),^12,13^ in molecular plasmas and in nuclear fusion. In addition, they are also driving the development of soft-ionization mass spectrometry techniques.^14^ They also govern the chemistry in space and planetary ionospheres^15,16^ affecting the transmission of radio and satellite signals.^16,17^

Moreover, ChemI are reactions of great interest, since they are characterized by a very special peculiarity. When the energetic reagent X* inducing ionization is a noble gas (Ng) excited in its first electronic metastable level (Ng*) (e.g., by collisions with energetic electrons or photons and/or by the interaction with cosmic rays), it overcomes its usual inertness, gaining a very high chemical reactivity. Indeed, Ngs* become extremely reactive, since they are excited open-shell atoms showing lifetime and energy content sufficient to ionize most atomic or molecular partners M by two-body collisions, forming parent (M^+^) or aggregate (NgM)^+^ ions accompanied by the spontaneous emission of electrons.^8−10,18−22^ Moreover, in the case of molecular targets, ionic products can include also results of rearrangement and dissociative phenomena.

It is well-known that ChemIs are controlled by an optical interaction potential (see next sections), whose real part drives the collision dynamics while its imaginary part, triggering the passage from neutral reagents to ionic products, accounts for the “opacity” of the system.^9,10^ Our group recently proposed^23−25^ a new theoretical approach to the study of ChemIs of Ne* reagent whose innovative aspects can be so summarized:

- Real and imaginary parts are not independent (as considered until now) but interconnected, since depending on the same components of the intermolecular forces involved and, according to their different nature, they are properly classified as chemical and physical interaction components.
- While the forces of chemical origin dominate at short separation distances between interacting partners, the physical ones prevail at intermediate and large distances.
- Direct and indirect reaction mechanisms are properly identified, being controlled by forces of chemical and physical nature, respectively. The switching between the two type of mechanisms is modulated by the strength and anisotropy of the intermolecular electric field associated with the interaction.
- The indirect mechanism includes also possible radiative contributions.^9,18,26,27^ In particular, in complexes formed at large separation distances by two-body collisions, the weak electric field generates non-adiabatic couplings between fine levels of the ^3^P_J_ atom^28^ and the coupling ^3^P_2,0_–^3^P_1_ stimulates an energy exchange through a “virtual” photon,^26^ emitted by the metastable atom and absorbed by the other partner. Note that the “virtual” term is adopted to distinguish this energy exchange process within an interacting system from canonical photoionization phenomena of isolated species.
- Appreciable polarization effects are stimulated as the separation distance further decreases, that are accompanied by a pronounced deformation of the “floppy” outer 3s electronic cloud of a metastable atom. Such a deformation can be described as a sort of 3sp and 3spd mixing (hybridization) that triggers the 3d–2p optical transition with again a “virtual” photon emission.^26^
- At shorter separation distances and under increased electric field conditions, chemical forces emerge that promote an exchange or direct mechanism with the passage of an electron from a HOMO orbital of M to the open-shell Ne^+^ ionic core accompanied by the 3s electron emission.

The recent application of our new theoretical approach to the simplest and basic Ne*(^3^P_2,0_) + Ar, Kr, and Xe reactions permitted us to point out that ChemIs effectively occur via two different microscopic mechanisms, whose relative importance depends on the quantum state of reagents and products and is modulated by kinetic energy *E*_c_ exploited and/or by interatomic (separation) distance *R* probed during each collision event.^23−25^ Reaction cross sections have been calculated in a synergic way for each |*J*, Ω⟩ → |*J*′, Ω′⟩ state selected reaction channel, where *J* and *J*′ are the total electronic angular momentum quantum number, defining also the spin orbit level of reagents and products, respectively, while Ω and Ω′ quantize the absolute projection of the **J** or **J′** vectors along **R**, for reagents and products, respectively. The validity test of predictions was provided by the internally consistent rationalization of experimental findings obtained in our^23−25^ and in other laboratories.^21,26,29−31^

Present interest is focused on other crucial details of the electronic rearrangements, with their effect on the reaction mechanism modulation. Particular attention is here addressed to the role of the orbital angular momentum of the collision complex that, determining the energy barrier due to the centrifugal potential accompanying all collision events, strongly modulates the range of mainly probed separation distances, especially under low collision energy. The results of the present investigation are considered by us as basic for the generalization of this new approach to ChemI involving molecules. In particular, the full characterization of atom–atom reactions can help to correctly identify all reaction channels in ChemI of molecules with their relative role just modulated both by the collision energy and by orbital angular momentum of the collision complexes.

## Methods: The Angular Momentum Hund Coupling Schemes for Chemi-Ionization Reaction Dynamics

An additional and innovative aspect of the proposed approach is to cast light on the sequence of angular momentum couplings that accompanies the dynamics of elementary atom–atom processes occurring via two-body collisions. In particular, since any collision represents a half rotation of the interacting diatomic complex, it must be confined in specific sequences of Hund’s cases, representing the proper scheme of total (electronic + nuclear rotation) angular momentum couplings/decouplings within the interatomic electric field, whose strength varies with *R*.^32,33^ It is useful to remember that all possible different situations are included in five Hund’s cases, indicated as (e), (d), (c), (b), and (a), the detailed description of which can be found in refs (32 and 33). However, if both reagents and products are open-shell atoms with sufficient high spin–orbit (SO) splitting, as the present ones, the collision dynamics is confined within a sequence of Hund’s cases (e), (c), and (a). As a consequence, the radiative mechanism is emerging in the passage from (e)–(c) cases, while the exchange mechanism becomes dominant during the (c)–(a) transition. Unique information on electronic rearrangements controlling the chemical reactivity under state selected conditions can be so extracted accounting for all details emerging from the angular momentum sequence adopted by the system.

The proposed approach^23−25^ is founded on the identification of the leading interaction components, which drive the collisions between closed and open-shell “P” atoms, and on their modeling in terms of fundamental chemical–physical properties of reagents and products, as electronic polarizability, ionization potential, and electron affinity. The collision dynamics and the reaction probability have been described adopting suitable formulations of such components. Accordingly, the interaction, controlling the collision dynamics in each entrance and exit channel, has been represented^24,25^ as a proper combination of an isotropic *V*_0_ and an anisotropic *V*_2_ component, both depending on *R*, with the SO splitting Δ. Moreover, while *V*_0_, determined by the balance of size repulsion with dispersion and induction attraction plus polarization effects, indirectly fixes the average interatomic electric field, *V*_2_ indirectly determines the anisotropy of the same field. In particular, the effectiveness of the *V*_2_ component, arising mostly from the selectivity of CT associated with the configuration interaction between states of the system with the same symmetry differing for an electron exchange, is dependent on the type and degree of valence orbital alignment within the interacting system. Therefore, it is also directly affecting the modulation of the reaction probability. On this ground, entrance and exit channels and TS are considered to form a unique manifold of quantum states accessible to the same system, confined into specific environments, whose features depend on a critical balance of the leading interaction components involved.

**Figure 1:**
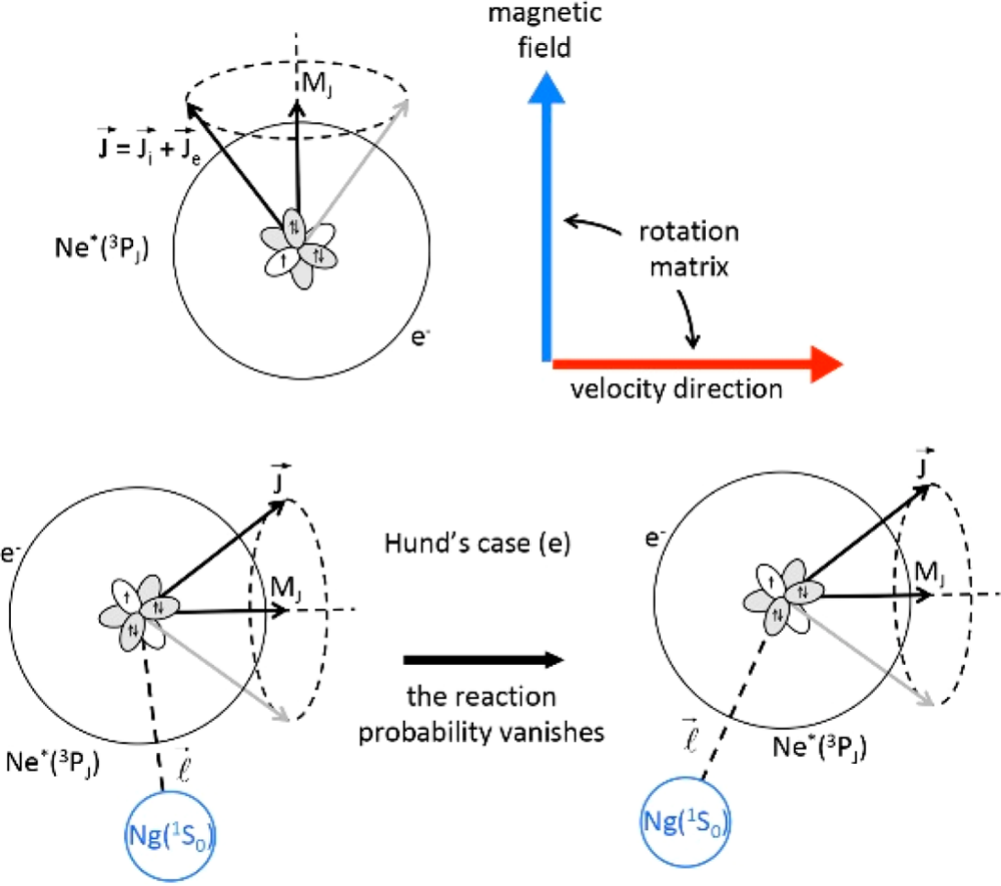
Upper panel: Total electronic angular momentum **J**, that arises as a sum of internal **J**_**i**_ and external **J**_**e**_ contributions, and its projection with respect to a quantization axis **z**, as that associated with an external magnetic field. Lower panel: Coupling schemes for Hund’s case (e), where the open-shell atom maintains its alignment with respect to a space fixed quantization axis since it is colliding at very large R (here the role of the interaction potential is negligible).  represents the collision orbital angular momentum, while *M*_*J*_ defines the projection of the **J** vector along a direction that can also be the one of an external field or of the atom velocity direction. The black color refers to Ne*, while the blue color indicates Ng.

**Figure 2:**
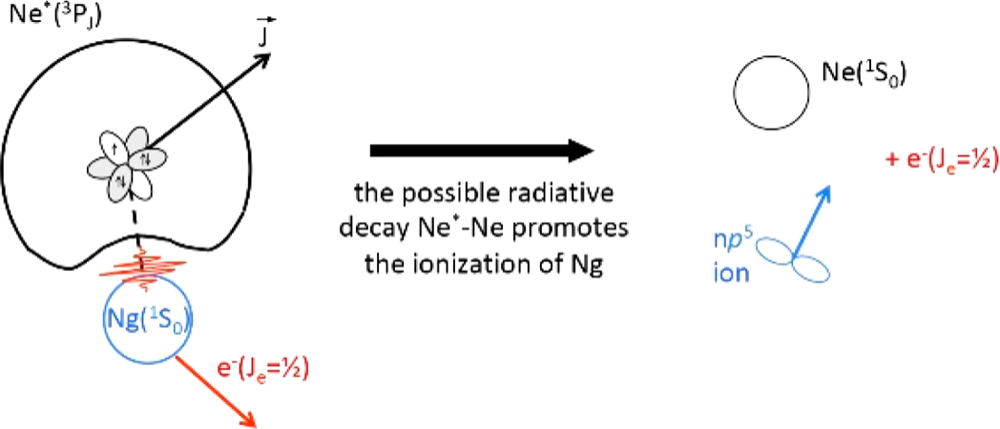
Coupling schemes for reactions occurring in the transition between Hund’s cases (e) and (c). The photoionization mechanism, promoted by optical transition of Ne* stimulated within the Ne*(^3^P_2,0_)–Ng collision complex (Ng = Ar, Kr, Xe), is also depicted. In all cases, the black color refers to Ne* and Ne, while the blue color indicates Ng and Ng^+^.

**Figure 3:**
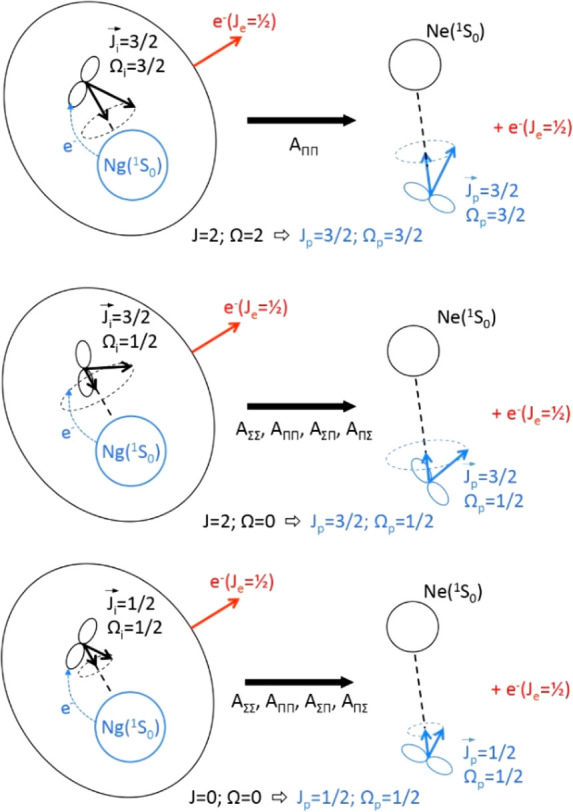
Representation of reactions occurring along three different channels, promoted by Ne*(^3^P_2,0_)–Ng collisions (Ng = Ar, Kr, Xe) with angular momentum coupling confined in Hund’s case (c), where radiative (physical) and exchange (chemical-oxidation) mechanisms are competitive. The three different channels are identified by the use of proper quantum numbers, typical of a diatomic interacting complex. The quantization axis coincides with the interatomic field direction, and *A*_ΛΛ′_ represents the coupling term between the entrance and exit channels defined according to guidelines recently reported in refs (23−25). In all cases, the black color refers to Ne* and Ne, while the blue color indicates Ng and Ng^+^.

**Figure 4:**
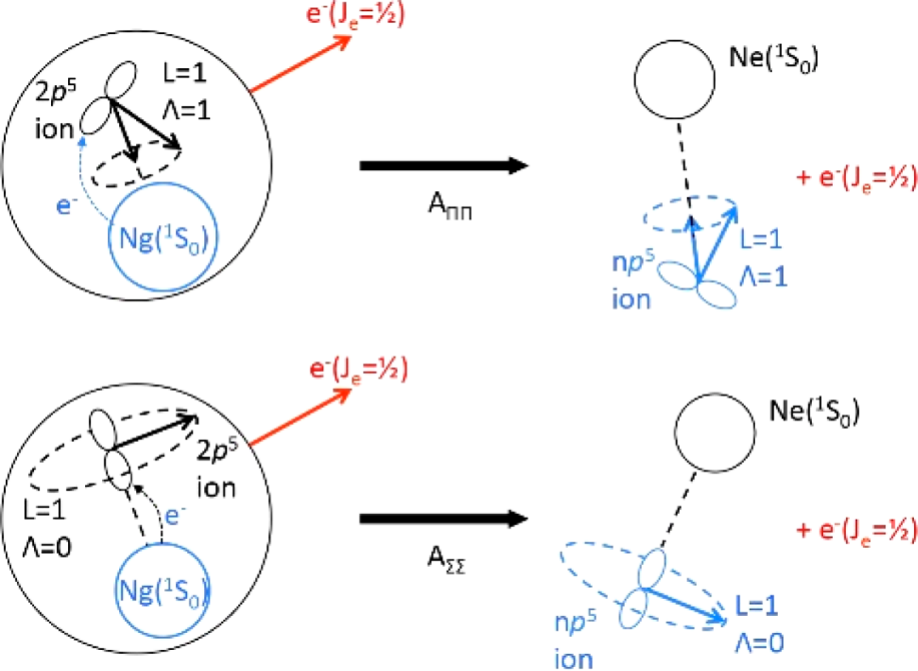
Representation of reactions occurring along two basic different channels, promoted by Ne*(^3^P_2,0_)–Ng collisions (Ng = Ar, Kr, Xe) with angular momentum coupling confined in Hund’s case (a). The exchange (chemical-oxidation) mechanism becomes dominant here since emerging in the passage from Hund’s (c)–(a) cases. The two different channels are identified by the use of proper molecular quantum numbers. The quantization axis coincides with the interatomic field direction, and *A*_ΛΛ′_ represent the coupling terms between the entrance and exit channels defined according to guidelines recently reported in refs (23−25). In all cases, the black color refers to Ne* and Ne, while the blue color indicates Ng and Ng^+^.

The proposed approach, exploiting the complete representation of the interaction components presented above, directly leads (see below) to identifying the sequence of angular momentum couplings that accompanies the dynamics of two-body collisions along each reaction channel. The characterization of this sequence provides further unique information on the selectivity of electronic rearrangements, which trigger electron transfer and ejection with their probability modulated by the type and degree of valence orbital alignment within the collision complex. The related selectivity controls indeed the microscopic evolution along each reaction channel, associated with a specific quantum state of reagents and products. Since any collision represents a half rotation at the *R* variable of the interacting complex, it must be confined in a specific sequence of Hund’s cases, representing the proper scheme of angular momentum couplings operative within any formed adduct. Specifically, for a freely flying reagent, the total electronic angular momentum **J**, enclosing external **J**_**e**_ and internal **J**_**i**_ contributions, each one determined as coupling of orbital **L** and spin **S** components, assumes projections defined by the quantum number *M*_*J*_ with respect to a quantization axis (along *z*) as that, for instance, of an external magnetic field (see upper panel of Figure 1). Instead, nuclear-rotation contributions arise exclusively during the collisions since they depend on the orbital angular momentum  of the rotating complex (see lower panel of Figure 1). The type of resulting coupling is directly controlled by the strength and anisotropy of the interatomic electric field, related to *V*_0_ and *V*_2_ components and that are varying with *R*. If both reagents and products are open-shell atoms with sufficient high SO splitting, as the present ones, the angular momentum , that triggers Coriolis (centrifugal) effects, plays a role of some relevance essentially at large *R*, where the interaction SO is dominant, with the role of the interaction potential being strongly reduced, providing only very weak perturbations. Under such conditions, the system is confined in the Hund’s case (e), as depicted in Figure 1, where **J** tends to maintain its projection *M*_*J*_. At shorter *R*, where the interaction components emerge,  accounts only for an average effect on the centrifugal barrier and the selectivity of the collision dynamics, determined exclusively by electronic angular momentum couplings, is confined within a sequence of Hund’s cases (c) and (a). Specifically, here the proper quantization axis becomes that of the interatomic electric field associated with the interaction potential. Under such conditions, the competition emerges between the **L** + **S** → **J** couplings, leading to spin–orbit levels, and the direct coupling of **L**, decoupled from **S**, with the interatomic electric field, leading to molecular states of defined symmetry, represented by the quantum number Λ (the absolute projection of **L** along **R**). The critical balance of the interaction terms involved is then crucial to assess what is the dominant Hund’s case and where a transition between two different situations occurs. According to the ample phenomenology of “P” atom interaction, recently summarized in refs (24 and 25), and taking into account the strength of the anisotropic interaction component *V*_2_ and the value SO splitting (Δ),^24,25^ it is possible to distinguish the following cases concerning the role of angular momentum couplings on the reactivity. They emerge as the probed *R* region decreases or the involved *E*_c_ increases:

- If |*V*_2_| ≪ Δ, the transition between Hund’s case (e) and Hund’s case (c) emerges, and here, polarization of external electronic clouds, Coriolis and SO effects play the major role. Moreover, the *indirect* mechanism, including radiative effects, is dominant (see Figure 2).
- The condition |*V*_2_| < Δ determines the Hund’s case (c) where a diatomic complex, formed by collision, emphasizes the role of **J**_**i**_ that aligns along **R** with projections defined by the quantum number Ω_i_ (see Figure 3). Here, the SO coupling determines a mixture of molecular states of different symmetry, whose relative weight varies with *J*_i_ and Ω_i_. Here, the two microscopic reaction mechanisms, *indirect* radiative and *direct* exchange (oxidation), become competitive.
- When |*V*_2_| ≅ Δ, the transition between Hund’s case (c) and Hund’s case (a) occurs and the *direct* exchange (oxidation) mechanism starts to dominate over the *indirect* (radiative) ones (see Figure 3).
- |*V*_2_| > Δ corresponds to Hund’s case (a) where a true diatomic molecule is formed with symmetry Σ (Λ = 0) or Π (Λ = 1) and this situation corresponds to a defined alignment of half-filled valence orbital within the collision complex, that is along **R** (see Figure 4), whose type depends on the involved reaction channel: here, the direct exchange (oxidative) mechanism is the dominant one.

Two important aspects must be pointed out for a proper assignment of the angular momentum coupling within each collision complex: (i) it is fundamental to know in detail the interaction potential and its anisotropy in a wide *R* range; (ii) while the anisotropic interaction component *V*_2_ shows at each *R* a defined strength related to the configuration interaction between states of the same symmetry coupled by CT, the sign is opposite for entrance and exit channels, since it generates bonding and antibonding effects. Moreover, since the isotropic interaction component and SO vary in entrance and exit channels, the situation of angular momentum coupling can be different in the two situations.

Unique information on electronic rearrangements controlling the chemical reactivity under state selected conditions can be so extracted accounting for all details emerging from the sequence probed by the system under the various conditions. As emphasized above, the radiative mechanism is emerging in the passage from (e) to (c) cases, while the exchange mechanism becomes dominant during the (c)–(a) transition.

## Results and Discussion on General Features of Reaction Mechanisms

The proposed method removes some limitations and inconsistencies of previous treatments^8−10,18,34,35^ and provides other basic information on the reaction dynamics. In particular:

(i) The potential energy curves (PECs), driving collisions in entrance and exit channels, belong to the same manifold of adiabatic states accessible to the system. While the strength of the interaction anisotropy is comparable in absolute scale with estimations by Gregor and Siska,^26^ for entrance, its sign is reverse. This is due to the fact that, for the (Ne···Ng)^+^ ionic core, the states correlating with Ne^+^ are those at higher energy, while states leading to Ne–Ng^+^ are the lowest ones. The configuration interaction, coupling states of the same symmetry, provides weak bonding effects by CT in the lowest states and weak antibonding effects in the highest ones. The sequence of states, with related PECs here obtained, is reverse to that previously published^26^ and extensively used in several papers.^36^ The present results are consistent with those provided by Dehmer^37^ and by Reed et al.^38^ with the phenomenology of several aggregates involving open-shell species as halogen atoms (isoelectronic with Ng^+^).^39^

(ii) Recently, in pioneering experiments^40^ involving a state selected Ne*(^3^P_2_) beam, the single occupied orbital on Ne* is considered as oriented along the interatomic axis (corresponding to Ω = 2). Our theoretical approach clarifies that this statement is inconsistent with the symmetry of the formed molecular state which shows a pure Π character at all distances (for which the quantum number Λ is equal to 1). For 2p^5^3s^1^ configurations of Ne*, the state *J* = 2, Ω = 2 (leading to ionic core in *J* = 3/2, Ω = 3/2) shows the single occupied p orbital aligned perpendicularly on the interatomic axis: this alignment determines a less repulsive effect, due to the configuration interaction, according to the pure Π character of the formed adduct.^23−25,37,39^

(iii) Real *V* and imaginary Γ parts of the optical potential *W*
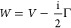
1control, respectively, approach/removal of reagents/products and transition probability from neutral entrance to ionic exit channels.^8−10,18,34,35^ They are usually considered and modeled as two independent interaction components. In several studies, they have been obtained from different sources and often scaled on the experimental results.^30^ Instead, as suggested by our approach, the two parts must be considered interdependent, since they arising from *adiabatic* and *non-adiabatic effects* associated with electron rearrangements (polarization, exchange, change in the angular momentum coupling, etc.) occurring within the interacting collision complexes.^23−25^ In other words, they relate to the quantum effects of the same system confined in a specific environment.

(iv) In the past, two different mechanisms, radiative and exchange, have been introduced.^18,26,34,35^ Our approach includes properly the two cases within the competition of direct and indirect processes, indicating *R* regions, or *E*_c_ values, where they are emerging. In particular, the basic markers are two coefficients, denoted *C*_*x*_ (for entrance) and *C*_*y*_ (for exit), representing the Σ molecular character degree of the PECs, with their dependence on *R* and on the considered channel.^23−25^ Specifically, where such coefficients maintain their asymptotic values, the interatomic interaction is vanishing and the reaction does not occur. In the long-intermediate *R* region, where *C*_*x*_ and *C*_*y*_ are slowly varying, the SO coupling, which controls the validity of the optic selection rules, is perturbed, losing partially or totally their validity with the emergence of radiative effects via a pure physical phenomenon.^25^ At shorter distances, the increased interatomic electric field destroys the SO coupling and *C*_*x*_ and *C*_*y*_ are strongly varying. In such an *R* region, here confined between 2.5 and 3 Å, the present systems assume a true Σ and Π molecular character and the exchange or direct mechanism becomes dominant, promoting a chemical oxidation process.^25^

(v) Under subthermal conditions (*cold chemistry*, for temperature *T* ≤ 10 K, *E*_c_ ≤ 0.1 kJ/mol), the radiative (photoionization) mechanism dominates; under hyperthermal conditions (*hot chemistry*, *T* ∼ 10^3^–10^4^ K, *E*_c_ ∼ 10–100 kJ/mol), reactions occur exclusively with the exchange (oxidation) mechanism; under thermal conditions (*T* ∼ 10^2^–10^3^ K, *E*_c_ ∼ 1–10 kJ/mol), the two mechanisms compete.

## Conclusions and Perspectives toward Molecular Systems

The investigation of atom–atom ChemI has been crucial to identify two basic reaction mechanisms, with their dependence on the state-to-state channel and their modulation by *E*_c_ or *T*. The present study focused on the selective role of the angular momentum couplings within the collision complexes formed under various conditions. The obtained results represent the proper starting point to rationalize and control the microscopic dynamics of more complex reactions. In the generalization of the methodology, a full description of ChemI involving molecules is an exciting challenge. This important target is pursued through the achievement of several intermediate steps. They include the correct identification of the nature and role of the intermolecular forces involved, the role of the electronic and nuclear rotation (centrifugal) angular momentum couplings, and, finally, the combined effect of atomic alignment and molecular orientation on the basic features of TS. Work is in progress in our laboratory, giving the opportunity to point out, in terms of old and new basic chemical aspects, differences between the chemistry of combustions and flames and that of terrestrial and interstellar environments characterized by hyperthermal, thermal, and subthermal regimes, respectively. Finally, our research group is working to investigate the basic chemical–physical principles that supervise the oxidation phenomena in elementary reactions that depend on the intermolecular forces determining the TS existence.
